# Hypergonadotropic hypogonadism and chromosomal aberrations: clinical heterogeneity and implications on the health of elderly men, case series

**DOI:** 10.1186/s12902-023-01359-6

**Published:** 2023-05-17

**Authors:** Tarik Elhadd, Ahmad Majzoub, Charlotte Wilson, Laura McCreight, Muna S. Mohamed, Fiona C. Green, Andrew J. Collier

**Affiliations:** 1 Department of Medicine, Qatar Metabolic Institute, Hamad Medical Corporation, Doha, Qatar; 2 Department of Urology, Hamad Medical Corporation, Doha, Qatar; 3 Department of Clinical Urology, Weill Cornell Medicine -Qatar, Doha, Qatar; 4 Department of Medicine, Ayr Hospital, South Ayrshire, Scotland; 5 Department of Medicine, Ninewells Hospital & Medical School, Dundee, Scotland; 6 Department of Medicine, Dumfries and Galloway Royal Infirmary, Dumfries, Scotland

**Keywords:** Male hypogonadism, Chromosomal abnormalities, Elderly, Hypergonadotropic hypogonadism

## Abstract

**Background:**

Hypogonadism in older men is often considered as late onset hypogonadism. However, this clinical condition results from primary testicular failure which could be of genetic origin with Klinefelter syndrome being the most common chromosomal abnormality associated with it.

**Case presentation:**

We report a heterogeneous group of cases who were diagnosed with hypergonadotropic hypogonadism in their adulthood and were found to have rare chromosomal aberrations. All were elderly men (in their 70 s and 80 s) for whom the diagnosis was made during the evaluation of incidental symptoms suggestive of endocrinopathy. The first had hyponatremia; the other two had gynaecomastia and features of hypogonadism noted during admission for various acute medical problems. With respect to their genetic results; the first had a male karyotype with balanced reciprocal translocation between the long arm of chromosome 4 and the short arm of chromosome 7. The second case had a male karotype with one normal X chromosome and an isochrome for the short arm of the Y chromosome. The third case was an XX male with unbalanced translocation between the X & Y chromosomes with retention of the SRY locus.

**Conclusion:**

Hypergonadotrophic hypogonadism in the elderly, may be due to chromosomal aberrations, resulting in heterogeneous and diverse clinical phenotypes. Vigilance must be exercised when seeing cases with subtle clinical findings. This report suggests that in selected cases of adult hypergonadotropic hypogonadism, chromosomal analysis may be indicated.

## Introduction

In females the process of menopause is well established. However, in males the process of age–related decline in sex hormones is more subtle and less abrupt. Male hypergonadotropic hypogonadism when detected in late adulthood may be diagnosed as late onset hypogonadism, even in the absence of specific symptoms. The decline in testosterone and rise in gonadotrophins is thought to represent a normal aging process in many males [1, 2]. Many patients with mild hypogonadism may be asymptomatic or may have mild non-specific symptoms. As such the diagnosis may be delayed and picked up during an investigation of an unrelated medical condition or for a complication of chronic hypogonadism. On the genetic side, hypergonadotropic hypogonadism is classically attributed to primary testicular failure due to Kleinfelter’s Syndrome (where the dysmorphic features coincide with the hypogonadism) [3]. Other non-genetic causes for hypergonadotropic hypogonadism include infections and inflammations of the testis such as mumps orchitis, testicular trauma, cryptorchidism, cancer and chemo/radiation therapy [4]. Advances in molecular genetics have revolutionized our understanding of genetic causes of hypogonadism by delineating an array of some unusual chromosomal aberrations that result in either infertility or hypogonadism or both. This report describes the presentation and investigation of three male patients who, for a variety of reasons, were subsequently found to have hypergonadotropic hypogonadism due to different chromosomal abnormalities. We will also discuss the implications for the clinicians.

### Case 1

A 74 year-old phenotypically normal male was admitted in Jan 2009, following a fall. His past medical history included chronic bronchitis, osteoarthritis, hypertension, recurrent falls and hyponatremia which had not been investigated earlier. The patient gave a history of excessive smoking while young, although he had stopped many years prior to this admission. There were no relevant symptoms on systemic enquiries. On clinical assessment; he was found to be euvolaemic normotensive with a BP 133/84 mmHg with random blood glucose of 4.8 mmol/l (86.4 mg/dl). On examination he was noted to have bilateral gynacomastia and infantile testes. Chest X-ray reported lung fibrosis and a possible fractured vertebra. Blood tests revealed hyponatremia with Na^+^ of 127 mmol/l, (Normal range 135-146 mmol/l) and normal K^+^, urea, creatinine, and liver function tests. Inflammatory markers were elevated (white cell count 18.8 X 10^9^/l, and CRP 110) with normal haemoglobin, platelets and haematological indices. The cause of raised inflammatory markers was thought to be either due to a urinary tract infection or the vertebral fracture. Further investigation of the pituitary hormones showed hypergonadotropic hypogonadism with LH 25.7 IU/L (Normal range 1–9 IU/L), FSH 23.9 IU/L (Normal range 1–12 IU/L) and low testosterone 6.5 nmol/L (Normal range 11–36 nmol/L), but otherwise normal pituitary function. The patient had a low plasma osmolality, normal urine osmolality, elevated urinary Na +. The hyponatremia was attributed to the Syndrome of Inappropriate ADH secretion (SIADH) due to chronic lung disease.

On finding the hypergonadotropic hypogonadism, further questioning revealed that the patient had not fathered any children but denied any erectile dysfunction. Blood was sent for chromosomal analysis and showed the following Karyotype: 46, XY,t(4;7)(q21.3;p15.3). The accompanying report stated that ‘*chromosomal analysis reveals a male karyotype with an apparently balanced reciprocal translocation between the long arm of one chromosome 4 and the short arm of one chromosome 7, in all cells examined. This in carriers of such translocation shall be associated with infertility, but in some cases even of the same pedigree such fertility problem may not be encountered.* The patient’s hyponatremia was managed appropriately. He was offered testosterone replacement which he declined.

### Case 2

An 87 year old man was referred to the medical receiving ward by his primary care physician who noted anaemia and worsening chronic renal impairment. The patient was asymptomatic. The patient’s only other hospital admission had been two years previously due to a fall. Investigation revealed a Na^+^ 148 mmol/l (Normal range 135-146 mmol/l), K + 5.2 mmol/l (Normal range 3.6–5.2 mmol/L), urea 22 mmol/L (Normal range 2.1–8.5 mmol/L), creatinine 225 µmol/l (Normal range 52.2–100 µmol/l), normal liver and bone profile. Full blood count confirmed a microcytic anaemia, with Hb of 6.5 g/dL (Normal range: 12.5–16 g/dL) and mean corpuscular volume 77 fl (Normal range 80–100 fl), mean corpuscular hemoglobin 23.2 pg (Normal range 24–34 pg), mean corpuscular hemoglobin concentration 36.9 g/dL (Normal range 33–36 g/dL), with normal white blood cell and platelets count. The serum iron was 6 µmol/L (Normal range:10.74 to 30.43 µmol/L), and ferritin was 134 ng/ml (Normal range: 24–336 ng/ml) with normal vitamin B12 and folate levels. Initial management was aimed at improving his renal impairment. Intravenous fluids were commenced and the patient underwent a blood transfusion. The patient’s height was 158 cm and he was noted to have a phenotype suggestive of hypogonadism with high pitched voice, bilateral gynaecomastia, infantile genitalia (small penis, testicular volumes 5-8 ml each, with the left being retractile), and little pubic, chest or axillary hair; questioning revealed that he had not been married or fathered any children. Both his siblings, a brother and sister- had married, but neither had children. In light of the above, a full endocrine assessment was undertaken. The TSH was 6.79 mU/L (Normal range 0.3–6 mU/L) and fT4 was 9.5 pmol/L (Normal range 9–24 pmol/L); this may well be normal for the patient as TSH tends to rise with age, rather than indicating subclinical hypothyroidism. The patient also had very low testosterone of 1.2 nmol/l (Normal range 11–36 nmol/L), elevated FSH 34.1 IU/L (Normal range 1–12 IU/L) and LH 32.8 IU/L (Normal range 1–9 IU/L). Other pituitary hormones were normal.

Due to the hormone profile, family history and the patient phenotype, chromosomal analysis was carried out and this showed the following Karyotyp*e; 46,X,i(Y)(p10).ish i(Y)(p)(DXYS129* +  + *,DYZ3,DXYS61*-). The impression of the genetic evaluation was as follows: *‘Chromosome analysis reveals a male karotype with one normal X chromosome and an isochrome for the short arm of the Y chromosome, in all cells examined. FISH studies using Y chromosome specific probes confirm this finding, this is an unbalanced karotype and would explain the patient’s phenotype’.* The results were explained to the patient who declined hormonal therapy. He was discharged following improvement of his clinical condition.

### Case 3

A 76 year old gentleman with a history of chronic bronchitis was admitted with left chest pain and productive cough. He was diagnosed with acute exacerbation of chronic bronchitis. He gave a history of right breast carcinoma which was resected several years before his admission. His physical examination revealed low grade fever (37.5 °C). He had a short stature (157 cm), smooth skin and high pitched voice. His genital examination revealed a normal sized phallus but with bilateral atrophic testes. On questioning he was found to have a history of infertility, which was never investigated, without any sexual dysfunction. Investigation confirmed hypergonadotropic hypogonadism with an FSH 27.8 IU/L (Normal range 1–12 IU/L), LH 21 IU/L (Normal range 1–9 IU/L) and serum testosterone 0.9 nmol/L (Normal range 11–36 nmol/L). His chromosomal analysis confirmed an XX male with unbalanced translocation between the short arm of chromosome X and the Y chromosome with normal SRY positive locus. Further details on this case have been reported elsewhere [5]**.**

## Discussion and conclusions

Primary gonadal failure leads to impaired secretion of gonadal steroids, namely testosterone. This causes elevated FSH and LH levels as the negative feedback loop is decreased. The chromosomal abnormalities leading to hypergonadotropic hypogonadism are generally caused by specific syndromes, which are generally rare and most are associated with typical dysmorphic features. The most common of these is Klinefelter Syndrome, which has an incidence of 1 in 1,000 males [6]. Other less common causes include: chromatin negative gonadal dysgenesis; anorchidia; Noonan Syndrome; abnormal testosterone synthesis; agenesis of Leydig cells; Sertoli Cell Only Syndrome (SCO); Smith-Lemli-Opitz Syndrome; Steinert myotonia and Bloom Syndrome. A more complete list of causes can be found in a text published by Topaloglu [7]. Furthermore, chromosomal aberrations involving either the sex chromosomes or autosomes can also result in hypogonadotropic hypogonadism [8–10], particularly Kallman syndrome [11, 12]. Hypergonadotropic hypogonadism discovered in middle or old age is generally ascribed to late onset hypogonadism.

The first two cases demonstrate that some patients may indeed harbour unusual chromosomal aberrations which may not usually be associated with any dysmorhic features (Table 1). The presence of gynaecomastia, which is not an uncommon finding in elderly males, may alert clinicians to rather unusual causes. Furthermore, good history taking is of paramount importance to point to ‘something other than simply late onset hypogonadism’.

**Table 1:** Characteristics of the included cases

Cases	Age	Presentation	Hormone profile	Genetic profile
Case 1	74 years	Recurrent syncope	Testosterone = 6.5 nmol/L FSH = 23.9 IU/L LH = 25.7 IU/L	46, XY, t(4;7)(q21.3;p15.3)
Case 2	87 years	Complications of CKD	Testosterone = 1.2 nmol/L FSH = 34.1 IU/L LH = 32.8 IU/L	46,X,i(Y)(p10).ish i(Y)(p)(DXYS129 + + ,DYZ3,DXYS61-)
Case 3	76 years	Recurrent chest pain	Testosterone = 0.9 nmol/L FSH = 28 IU/L LH = 21 IU/L	46 XX.ish der(X)t(X;Y)(p22.3;p11.2)(SRY)

Reference ranges: Testosterone 11–36 nmol/L, LH 1–9 IU/L, FSH 1–12 IU/L

*LH* Luteinizing hormone; FSH: Follicular stimulating hormone

Cases of hypergonadotropic hypogonadism associated with chromosomal aberrations are rarely reported in the literature. Zahed et al. (2004) described ring chromosome 18q and jumping translocation 18p in an adult with hypergonadotropic hypogonadism [13]. As with case 1, this patient had a reciprocal translocation which is typically defined by the exchange of genetic material between two non-homologous chromosomes. As no loss or gain of genetic material is observed, patients typically have no identifiable phenotypic alterations. However, these patients may produce unbalanced chromosomal translocations during spermatogenesis making them prone to infertility, recurrent miscarriage or delivery of offspring with genetic abnormalities. We found one other report of a patient with hypergonadotropic hypogonadism involving chromosome 4, on Medline [14]. In this case the authors suggested 3 candidate genes, two of which were situated on 4q (located on 4q28.3 and 4q31.2).

**Fig. 1:**
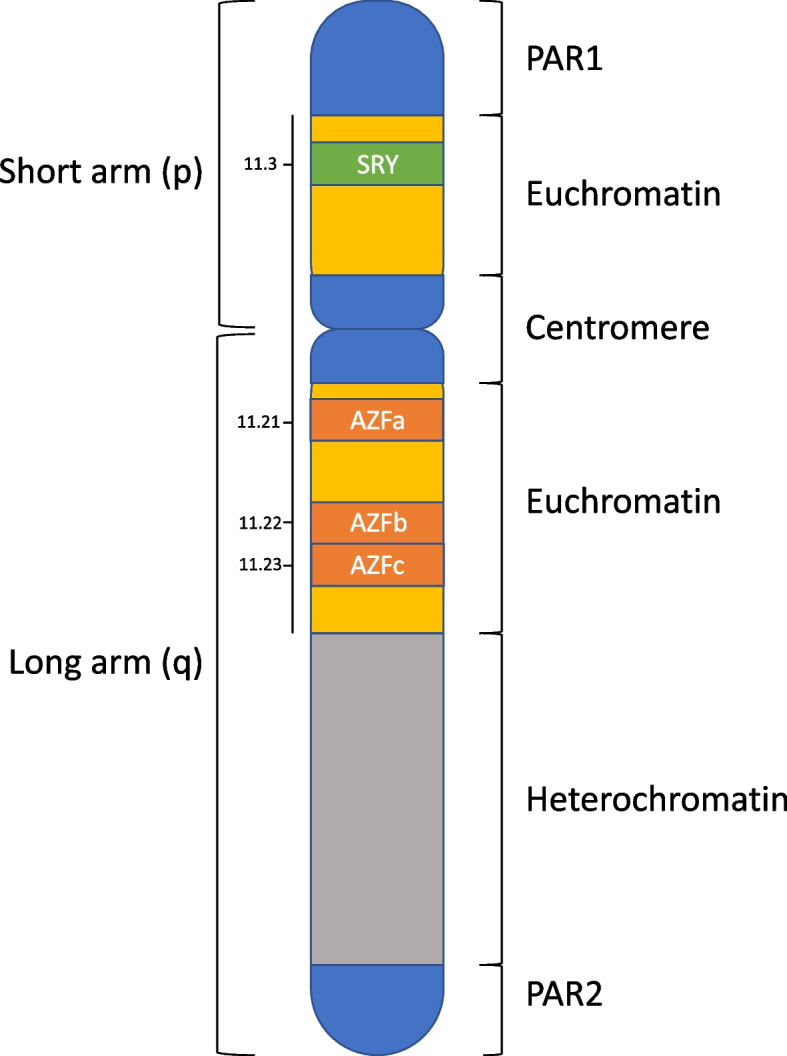
Distribution of important regions along the Y chromosome

In case 2, an unbalanced translocation of the Y chromosome was detected and so the resulting phenotype abnormality was perhaps less surprising. Polymerase chain reaction (PCR) amplification of the Y chromosome is commonly performed to look for deletions in the azoospermia factor regions of its long arm (Yq) in men with severe male infertility. Such deletions have been reported in up to 23% of infertile men with azoospermia [15]. Nonetheless, structural changes or deletions along the short arm of the Y chromosome are much less frequently reported. The short arm contains the pseudo-autosomal region 1 (PAR1) which, as its name suggests, has the genetic behaviour of an autosome (Fig. 1). Its genes are involved in mental and stature development [16]. Additionally, the short arm contains an euchromatic region that carries several genes the most important of which is the sex determining region on Y (SRY). Our patient had an isochromosome of the short arm and this structural abnormality can occur during mitosis or meiosis and results from either centromere misdivision or U type strand exchange [17]. Such an aberration can occur in any acrocentric chromosome but is more commonly reported in sex chromosomes (both X and Y). X isochromosomes has been most commonly associated with Turner syndrome occurring in about 15% of cases [17]. On the other hand, Y isochromosomes have been much less frequently reported and may be associated with male or female phenotypes [18, 19]. Male patients presenting with such chromosome aberration have typically been described to have primary testicular failure with variable phenotypic features of hypogonadism.

Case 3 was found to have a 46,XX Karyotype. This disorder of sex development is a rare condition believed to have an incidence of 1:20,000 males [20]. A variable mismatch between the patients’ genotypic and phenotypic features occur, and they may present at an earlier age because of ambiguous genitalia or may have a completely normal masculinity and present at a later age seeking fertility [21]. The SRY gene is preserved in such individuals and this is why they maintain a male phenotype. In 80% of cases the SRY gene is translocated into an X chromosome or an autosome and in the remaining 20%, hidden mosaicism for the SRY gene has been postulated [22]. In their case series and literature review, Majzoub et al. reported 55 cases of 46, XX men and described their overall features [23]. All patients had testicular atrophy and their histopathology showed Sertoli cell only syndrome and Leydig cell hyperplasia. Hypergonadotropic hypogonadism is typically identified with a reported mean FSH level of 40.4 ± 22.2 IU/L, mean LH 23.4 ± 13.4 and mean testosterone 274.3 ± 135.3 ng/dl (9.5 ± 4.7 nmol/L). Features of hypogonadism including reduced hair distribution and gynecomastia were identified in 26.6% and 40% of the cases. From the fertility perspective, these patients are considered sterile as they lack all the AZF regions necessary for normal spermatogenesis and their options are limited to assisted reproduction using donor sperm or adoption.

In all three cases described above, their hypergonadotropic hypogonadism state occurred secondary to genetic aberrations and hence should not be misdiagnosed as standard cases of late onset hypogonadism. It is worth noting that patients who chronically suffer from hypogonadism, especially at an earlier age, may develop adaptation to the lower testosterone levels and may not experience the typical symptoms of hypogonadism seen in others. In such cases, the endocrine disturbance may be incidentally discovered at a later age and during work-up for an unrelated clinical condition. The clinician should therefore be aware of the vast array of chromosomal aberrations associated with hypergonadotropic hypogonadism, and consider chromosomal analysis in selected cases. It is also worth mentioning that none of our patients had evidence of significant osteoporosis and hence testosterone replacement to improve bone health was not warranted.

One may argue about the cost of testing for chromosomal abnormalities and whether this would be justified. Indeed we do not recommend chromosomal analysis testing in all cases of elderly hypergonadotropic hypogonadism. However, we argue that in the presence of subtle chronic symptoms of hypogonadism or a history of subfertility either in the patient or his family members, karyotyping may be justified. With the exception of Klinefelter syndrome or 46,XX male cases, in whom genetic testing may help in the prevention of health related consequences including higher likelihood of breast carcinoma, the identification of genetic abnormalities in other cases may not influence their treatment plan. Nonetheless, several virtues for testing can be attained and include: (1) finding an aetiology or explanation for the patients’ long standing symptoms; (2) guiding the evaluation of patients’ siblings and other younger family members who may carry the same genetic aberration and/or suffer from the same symptoms; (3) directing fertility treatment plans for patients seeking them through offering in vitro fertilization coupled with preimplantation genetic diagnosis whenever possible.

## Data Availability

The authors declare that data supporting the findings of this study are available within the article.

## Abbreviations

BP: Blood pressure
Na+: Sodium
K+: Potassium
CRP: C-Reactive protein
LH: Luteinizing hormone
FSH: Follicular stimulating hormone
ADH: Anti-diuretic hormone
SIADH: Syndrome of inappropriate ADH secretion
Hb: Hemoglobin
NR: Normal range
MCV: Mean corpuscular volume
MCH: Mean corpuscular hemoglobin
MCHC: Mean corpuscular hemoglobin concentration
WCC: White cell count
TSH: Thyroid stimulating hormone
fT4: Free thyroxin
GH: Growth hormone
IGF-1: Insulin like growth factor
FISH: Fluorescent in situ hybridization
PCR: Polymerase chain reaction
PAR1: Pseudo-autosomal region 1
SRY: Sex determining region on Y

## References

[1] Tserotas K, Merino G (1998). Andropause and the aging male. Arch Androl.

[2] Anawalt BD, Merriam GR (2001). Neuroendocrine aging in men. Andropause and somatopause. Endocrinol Metab Clin North Am.

[3] Forti G, Corona G, Vignozzi L, Krausz C, Maggi M (2010). Klinefelter's syndrome: a clinical and therapeutical update. Sex Dev.

[4] Viswanathan V, Eugster EA (2011). Etiology and treatment of hypogonadism in adolescents. Pediatr Clin North Am.

[5] Hado HS, Helmy SW, Klemm K, Miller P, Elhadd TA (2003). XX male: a rare cause of short stature, infertility, gynaecomastia and carcinoma of the breast. Int J Clin Pract.

[6] Arshad MA, Yamani MMAO, Elbardisi HT, Majzoub A, Parekattil SJ, Esteves SC, Agarwal A (2020). Novel Approaches in the Management of Klinefelter Syndrome. Male Infertility: Contemporary Clinical Approaches, Andrology, ART and Antioxidants.

[7] Topaloğlu AK (2017). Update on the genetics of idiopathic hypogonadotropic hypogonadism. J Clin Res Pediatr Endocrinol.

[8] Kikuchi I, Nagamine M, Ueda A, Mihara K, Seita M, Minoda M (1993). Chromosomal translocation t(13;16) in a patient with idiopathic hypogonadotropic hypogonadism. Intern Med.

[9] Ozalp O, Yilmaz Z, Kilicdag EB, Bolat F, Bagis T, Sahin FI (2006). 45, XY, der(13;14)(q10;q10) in an azoospermic man with hypogonadotrophic hypogonadism. Asian J Androl.

[10] Elbistan M, Aydin M, Bagci H, Kara N (1994). A case of hypogonadism with a translocation: t(4; 12) (q25; q24.2). Indian J Pediatr.

[11] Schinzel A, Lorda-Sanchez I, Binkert F, Carter NP, Bebb CE, Ferguson-Smith MA (1995). Kallmann syndrome in a boy with a t(1;10) translocation detected by reverse chromosome painting. J Med Genet.

[12] Casamassima AC, Wilmot PL, Vibert BK, Shapiro LR (1993). Kallmann syndrome associated with complex chromosome rearrangement. Am J Med Genet.

[13] Zahed L, Oreibi G, Azar C, Salti I (2004). Ring chromosome 18q and jumping translocation 18p in an adult male with hypergonadotrophic hypogonadism. Am J Med Genet A.

[14] Tzschach A, Ramel C, Kron A, Seipel B, Wüster C, Cordes U (2009). Hypergonadotropic hypogonadism in a patient with inv ins (2;4). Int J Androl.

[15] Majzoub A, Arafa M, Clemens H, Imperial J, Leisegang K, Khalafalla K (2022). A systemic review and meta-analysis exploring the predictors of sperm retrieval in patients with non-obstructive azoospermia and chromosomal abnormalities. Andrologia.

[16] Helena Mangs A, Morris BJ (2007). The Human Pseudoautosomal Region (PAR): origin function and future. Curr Genomics.

[17] Lorda-Sanchez I, Binkert F, Maechler M, Schinzel A (1991). A molecular study of X isochromosomes: parental origin, centromeric structure, and mechanisms of formation. Am J Hum Genet.

[18] Aftab A, Shankar K, Kar B (2020). Rare case of monocentric isochromosome Y with inversion duplication of p arm in patient diagnosed with azoospermia. Andrologia.

[19] Hemmat M, Hemmat O, Boyar FZ (2013). Isochromosome Yp and jumping translocation of Yq resulting in five cell lines in an infertile male: a case report and review of the literature. Mol Cytogenet.

[20] de la Chapelle A (1981). The etiology of maleness in XX men. Hum Genet.

[21] Vorona E, Zitzmann M, Gromoll J, Schüring AN, Nieschlag E (2007). Clinical, endocrinological, and epigenetic features of the 46, XX male syndrome, compared with 47, XXY Klinefelter patients. J Clin Endocrinol Metab.

[22] Rajender S, Rajani V, Gupta NJ, Chakravarty B, Singh L, Thangaraj K (2006). SRY-negative 46, XX male with normal genitals, complete masculinization and infertility. Mol Hum Reprod.

[23] Majzoub A, Arafa M, Starks C, Elbardisi H, Al Said S, Sabanegh E (2017). 46 XX karyotype during male fertility evaluation; case series and literature review. Asian J Androl.

